# Camptocormia due to myotinilopathy, Parkinson’s disease, or both? Camptocormia and axial myopathy

**DOI:** 10.1186/s42466-023-00276-2

**Published:** 2023-09-14

**Authors:** Josef Finsterer

**Affiliations:** Neurology & Neurophysiology Center, Postfach 20, 1180 Vienna, Austria

**Keywords:** MYOT, Parkinson’s disease, Camptocormia, Bent spine, Axial myopathy

Letter to the Editor.

We read with interest the article by Petry-Schmelzer et al. which is about a 78 year-old female with the double trouble, myofibrillar myopathy and Parkinson’s disease [1]. The first clinical manifestations at the age of 72 were camptocormia and leg weakness and were classified as onset of Parkinson’s disease [1]. A myofibrillar myopathy was diagnosed at the age of 78 at the earliest and attributed to the variant c.179 C > T in *MYOT* [1]. Unilateral resting tremor of the right hand has been interpreted as the second clinical manifestation of Parkinson’s disease [1]. Parkinson’s disease responded poorly to L-DOPA [1]. It was concluded that Parkinson’s disease is a rare differential diagnosis of camptocormia in patients with myofibrillar myopathy [1]. The study is impressive, but it has limitations that should be discussed.

We disagree with the interpretation of camptocormia as the first manifestation of Parkinson’s disease [1]. Myofibrillar myopathy, including myotilinopathy, can present as axial myopathy [2], and axial myopathy has been reported to present initially as camptocormia [3]. A strong argument for camptocormia being the first manifestation of myofibrillar myopathy but not Parkinson’s disease is that the patient developed leg weakness concomitant with camptocormia. Although the patient underwent lumbar spine surgery for vertebrostenosis at the age of 73, leg weakness did not improve. The first manifestation of Parkinson’s disease in the index patient was resting tremor. A strong argument for tremor as the initial manifestation of Parkinson’s disease is that tremor is a common manifestation and camptocormia a rare symptom of Parkinson disease. If a tear of the right supraspinatus muscle tendon occurred prior to the onset of camptocormia, this could also have been the first manifestation of myofibrillar myopathy. Since the patient presented at the age of 78 with a positive Trendelenburg sign [1], it can be assumed that a waddling gait was present, indicating proximal weakness. Was there evidence that this type of gait disturbance was already present at the age of 72?

We also disagree with the statement in the introduction that camptocormia occurs with a prevalence of 3–18% in patients with Parkinson’s disease [1]. In the Asian population, postural disorders, including camptocormia, have been reported in up to 27% of patients [4].

A limitation of the study is that it does not report at what point upper-limb weakness developed. The clinical neurologic examination at the age of 78 revealed quadriparesis, but there is no evidence of the onset or clinical manifestation of upper extremity weakness.

Myotilinopathy can also manifest as skeletal muscle pseudohypertrophy. Was there evidence of excessive muscle fat replacement in the index patient?

Myotilinopathy can manifest as a multisystem disease affecting various organs, including the heart [5]. We should know whether the index patient has been prospectively evaluated for cardiac involvement, which may manifest as dilated or hypertrophic cardiomyopathy [6] or arrhythmias [7], both of which may be associated with heart failure [8].

What is the explanation for the normal creatine-kinase in the index patient? Was it due to progressive conversion of muscle tissue into fat? Was the patient’s motor activity restricted due to quadriparesis or hypokinesia due to Parkinson’s disease?

In summary, the interesting study has limitations that call the results and their interpretation into question. Addressing these issues would strengthen the conclusions and could improve the status of the study. Before interpreting clinical presentations, a careful individual and family history must be taken and the patient must be carefully examined neurologically.

## Data Availability

All supporting data are available from te corresponding author.
